# Optical-Based Biosensors and Their Portable Healthcare Devices for Detecting and Monitoring Biomarkers in Body Fluids

**DOI:** 10.3390/diagnostics11071285

**Published:** 2021-07-16

**Authors:** Anh Tran Tam Pham, Angus Wallace, Xinyi Zhang, Damian Tohl, Hao Fu, Clarence Chuah, Karen J. Reynolds, Carolyn Ramsey, Youhong Tang

**Affiliations:** 1 Australia-China Science and Research Fund Joint Research Centre for Personal Health Technologies, Flinders University, Tonsley, SA 5042, Australia; anh.pham@flinders.edu.au (A.T.T.P.); angus.wallace@flinders.edu.au (A.W.); xinyi.zhang@flinders.edu.au (X.Z.); damian.tohl@flinders.edu.au (D.T.); hao.fu@flinders.edu.au (H.F.); karen.reynolds@flinders.edu.au (K.J.R.); carolyn.ramsey@flinders.edu.au (C.R.); 2 Medical Device Research Institute, Flinders University, Tonsley, SA 5042, Australia; clarence.chuah@flinders.edu.au

**Keywords:** biomarkers, biosensors, body fluids, optical detections, point-of-care, diagnostics, devices

## Abstract

The detection and monitoring of biomarkers in body fluids has been used to improve human healthcare activities for decades. In recent years, researchers have focused their attention on applying the point-of-care (POC) strategies into biomarker detection. The evolution of mobile technologies has allowed researchers to develop numerous portable medical devices that aim to deliver comparable results to clinical measurements. Among these, optical-based detection methods have been considered as one of the common and efficient ways to detect and monitor the presence of biomarkers in bodily fluids, and emerging aggregation-induced emission luminogens (AIEgens) with their distinct features are merging with portable medical devices. In this review, the detection methodologies that use optical measurements in the POC systems for the detection and monitoring of biomarkers in bodily fluids are compared, including colorimetry, fluorescence and chemiluminescence measurements. The current portable technologies, with or without the use of smartphones in device development, that are combined with optical biosensors for the detection and monitoring of biomarkers in body fluids, are also investigated. The review also discusses novel AIEgens used in the portable systems for the detection and monitoring of biomarkers in body fluid. Finally, the potential of future developments and the use of optical detection-based portable devices in healthcare activities are explored.

## 1. Introduction

The human body is mainly composed of water, and depending on the specific biological functions of each organ, water distribution varies throughout the body [1,2]. The water containing biological components inside the body is often called body fluid, and the biological analytes present in body fluid are called biomarkers, or biological markers [1,2,3]. One type of body fluid usually contains numerous biomarkers. For instance, human urine consists of urea, chloride, sodium and potassium salts, creatinine, and other substances; saliva contains sodium, calcium, phosphates, enzymes, mucins, and so on; blood and serum are comprised of glucose, copper, iron, white blood cells, red blood cells, albumin, cTnI, a prostate specific antigen, ascorbic acid, and so on [4,5]. Meanwhile, minerals, lactic acid, urea, electrolytes, nickel, lead, copper, iron, and zinc can be found in sweat [4,5]. Depending on the testing purpose, researchers can focus on collecting specific biomarkers through a desired body fluid. This kind of decision will have a significant effect on the biomarker evaluation. For many years, biomarkers have been considered one of the most critical components for monitoring patient health. They can be used to measure the state of patients, provide feedback for the effective application of treatment strategies, or evaluate everyday processes, pathogenic processes, or pharmacological responses to therapeutic intervention [6,7,8,9,10]. Although biomarkers are useful indicators of different biological processes, they can be difficult to measure and can lack sensitivity for the collection [11]. The potential solutions for supporting this type of monitoring are: (1) to enhance the quality of the output signals from the biomarker processing, and (2) to improve the quality of the signal collecting module.

Recently, the development of portable technology has shifted biomarker detection from traditional laboratory conditions to more portable user-friendly devices [12]. Many researchers have developed portable devices, equipped with various technologies, such as surface plasmon resonance (SPR), electrochemical assay, surface enhanced Raman spectroscopy (SERS), gel electrophoresis, etc., to provide a reliable operation and convenience for patients while achieving comparable results to laboratory measurements [12,13,14,15,16,17,18]. However, most of the techniques encounter issues such as a lack of accuracy, sensitivity, and specificity required for clinical diagnostic applications [15]. Amongst these portable devices, optical detection by colorimetry, fluorescence, or chemiluminescence methods, which can partially overcome those common issues, has become one of the most widely applied methods for identifying and monitoring biomarkers in body fluids [12,13,14,15,16,19,20].

This review is focused on portable devices that use mobile technology and three optical detection methods, including colorimetry, fluorescence, and chemiluminescence, to identify and measure biomarkers. First, an overview of the different optical detection methods is given. This is followed by a discussion of an existing portable device in which these optical methods are used for biomarker detection. The rest of the paper will discuss the development of the devices.

The aim of this review is to develop a decision matrix that can be applied when developing a new portable POC device to detect and measure biomarkers using optical methods.

## 2. Optical Detection Methods

Optical examination is based on the light response in the chemical or biochemical reactions between specific analytes and the agent probes. These methods exploit different chemical or biochemical effects to allow a measurement to be made, i.e., (1) colorimetric: colour changing of the final mixture; (2) chemiluminescent: a chemical reaction results in the mixture emitting photons; and (3) fluorescent/phosphorescent: activated molecules are excited by a specific wavelength light and are elevated from normal energy levels to higher energy levels. When they return to their original energy levels, the excess energy will be released in the form of photons, which create the fluorescence. Next, we give an overview of these three different optical detection methods, followed by a discussion of some general reports of portable devices in which these methods are used for biomarker detection. Device development is discussed in the rest of the sections.

### 2.1. Colorimetric

Colorimetric examination is based on observations of the colour changes caused by the chemical or biochemical reactions between the specific analytes and the agent probes [12,20,21,22]. When biomarkers and the colorimetric probes react, they absorb light in the visible range. Therefore, the advantage of this method is that operators can read the qualitative result with the naked eye, without bulky or expensive equipment [11,12,21,22,23]. Furthermore, quantitative measurements can also be carried out with compact processing devices such as colour sensors or simple image capture cameras, again avoiding cumbersome and costly equipment. Thanks to their advantages of being cost effective, easy to use and rapid, colorimetric methods are one of the most widely used applications in disease detection [12,13,14,15,16,19,20]. Depending on the specific biomarkers, the selective colorimetric probes can achieve the highest sensitivity in the detection of biomarkers at different concentrations.

Wang et al. [24] reported the development of the mobile device and microchip enzyme-linked immunoassays (ELISAs) using a colorimetric method to detect cancer biomarkers in body fluids. One process included in Wang’s report was the reliable detection of the human epididymis protein 4 (HE4) in urine which can be used as one of the biomarkers for ovarian cancer. To support HE4 detection, a specific ‘microchip’ is prepared to detect the protein HE4 in urine. In the measurement, the urine sample is loaded onto the microchip. After HE4 is physically captured on the microchip’s surface, a blocking substrate, bovine serum albumin (BSA), is added. After that, a detection antibody and a capture antibody are injected into the mixture to conjugate with HE4 to form an immune-complex, horseradish peroxidase (HRP). Finally, tetramethylbenzidine (TMB), as a substrate, is added to the HRP complex, resulting in the blue colour development [24]. A white light-emitting diode (LED) is used to supply the light for the imaging process. The colour image of the HE4 immuno-complex is captured by a mobile device camera or a charge-couple device (CCD), then analysed into red-green-blue (RGB) pixel values to give the correlation between the colour intensity and the level of HE4 in the urine samples. As a result, the sensitivity of the method was recorded as 19.5 ng/mL of HE4 concentration in urine and the measurement time was about 10 min. This colorimetric method is effective in detecting ovarian cancer by monitoring HE4 in urine samples [24].

In 2016, Dahl presented a portable urinalyser, consisting of a handheld computing device and a holder for the urine test strip, to detect multiple analytes in urine, such as leukocytes, nitrogen, uroalbumin, pH, bilirubin, and glucose [25]. Dahl used the standard urine strip from Diascreen 10, Arkay and a supporting plastic holder including 10 LEDs and 10 photo-sensors for each corresponding analyte on the test strip [25]. For the measurement, the urine strip is immersed in the urine sample container so that the fibre material on the strip can absorb a suitable urine amount for colour measurement. Then, the urine strip is loaded into the plastic reader with the sealed cap at the strip input port. The other end of the test strip reader connects to the smartphone through a standard USB or USB-C port, or the mic/headphone outputs. An app installed in the computing device manages the power supply for the strip reader to turn on the LEDs and activate the photo-sensors in the reader. The colour of the test strip changes depending on the presence of the analytes in the urine sample. In the smartphone app, a list of reference values for each biomarker is prepared following the standard from the strip manufacturer. When the colour intensity of one individual biomarker exceeds the norm, the app indicates the level of that chemical quantitively.

### 2.2. Luminescence

Luminescence is the emission of light from a substance which is not caused by the rising temperature. There are three main types of luminescence: fluorescence, phosphorescence, and chemiluminescence. Fluorescence and phosphorescence are types of photoluminescence while chemiluminescence has a different trigger condition for glowing [26]. Fluorescence and phosphorescence involve the electronic absorption of the molecules in a specific medium. In photoluminescence, molecules in a substance absorb photons of unique wavelengths and emit a photon of a longer wavelength in a critically short time [27]. The typical difference between fluorescence and phosphorescence is the glowing time; fluorescent molecules absorb the excitation photon in 10^−5^ s, internal conversion takes place in 10^−11^ s, and the lifetime of the fluorescence after the excitation source vanishes is around 10^−9^ s. In contrast, the phosphorescence process continues for up to several seconds after excitation [26]. Not all molecules can produce fluorescence; those that do have fluorescent properties that are often called fluorophores, or simply fluors [28]. Many researchers have shown that fluorescence is typically proportional to the number of biomolecules attached to fluorophores [14,15,22,27,29].

**Figure 1:**
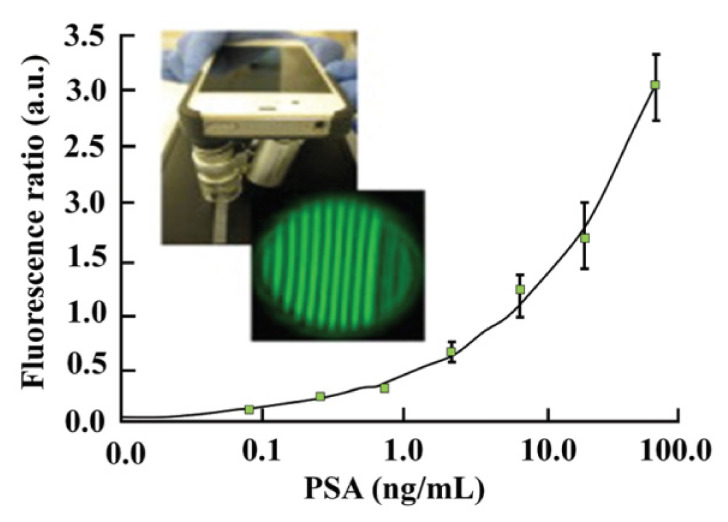
Nimse and co-workers used a portable smartphone-based fluorescence analyser to quantitively detect the prostate specific antigen (PSA) in the whole blood sample. The higher the PSA concentration is, the more intense the fluorescent signal is that is recorded [15].

In 2016, Nimse et al. [15] reported the application of a fluorescence method in the detection of the prostate-specific antigen (PSA) which supports the early detection of prostate cancer (PCa). Prostatic acid phosphatase was reported as the superior biological indicator for PCa where a PSA amount of over 4 ng/mL is considered to indicate the presence of cancer. As shown in Figure 1, Nimse and co-workers used colorimetry and fluorescence to detect PSA and reported that both methods could detect the early stage of PCa. Nevertheless, the low limit of detection in colorimetric immunoassays was 60 ng/mL, which was much higher than the lower LOD of the fluorescence method of 0.4–0.08 ng/mL in a whole blood sample. Hence, the fluorescence method has more advantages in PCa early detection than the colorimetric one [15].

Balwant and Kaur in 2016 reported a diagnostic method using the fluorescence principle on a test strip to detect sleep disorders in an individual by assessing a saliva sample [30]. In sleep disorders, there are some specific biomarkers which can be measured to estimate the disease condition, such as arginine, creatine, dihomo-linoleate, tyrosine, beta-endorphin, chromogranin A, linoleate, BDNF, and 3-methyl oxobutyrate [30]. In normal conditions, there is a small amount of these proteins in saliva. When these proteins are present in amounts exceeding the reference values, the patient is considered to have a sleep disorder. The saliva test strip is prepared with the selective agents for each target biomarker. The test accuracy was reported to range from 86% to 91.5% in a group of 65 patients.

In 2017, Gabr and Pigge used an aggregation induced emission (AIE) to detect the human serum albumin (HSA) in a urine sample [31]. The complex of Re(I) tricarbonyl is prepared from bis (pyridyl)- and bis(quinolyl) tetraarylethylene (TAE) ligands which were reported to enhance the fluorescent emission of the solution when combined with HSA. The final solution is excited by 396 nm ultraviolet (UV) light and emits light blue fluorescence with a wavelength of 509 nm. This method claimed to improve the LOD of HSA in a urine sample to 20 µM. In 2017, Zhang and co-workers [32] presented an assay using an AIE-active fluorophore (TPE-HPro) to detect L-Lactate oxidase (LOx) in aqueous fluid. This assay was excited at 373 nm wavelength and yielded the 570 nm green emission. The fluorescent signal lasted around 60 min, facilitating accurate measurement. The limitation of detection was reported to be as low as 5.5 µM. This research provides a potential for applying AIE in tracking the presence of L-Lactate in saliva and urine samples. Meanwhile, Zhang et al. [4] reviewed the constituents and clinical biomarkers in urine, saliva, and sweat, and the role of currently developed AIE bioprobes that can quantitatively detect disease-related biomarkers in these biofluids. The review also highlighted that several applications of AIE bioprobes, such as paper-based strips and portable devices, are currently under development, and have the potential to be realised simply by use with smartphone data capture and analysis, and data cloud storage.

In contrast to fluorescence and phosphorescence, which require external excitation by light, chemiluminescence results from the chemicals reacting with each other [12,26,33]. In most chemical reactions, the excess energy is discarded as exothermic energy; however, in certain cases, some reactions result in the product having an electronically excited state. If the excited product relaxes by emitting photons and causing luminescence, it is called chemiluminescence [26]. Compared with fluorescence, chemiluminescence has more potential for application for portable analysis purposes because it does not require external excitation, which reduces the complexity of the device design and fabrication [12,26]. Chemiluminescence detection has been applied in the tracking of cancer biomarkers for many years. Dimitrakov and Buffington [34] described a microfluidic chemiluminescence ELISA using paper strips to detect the biomarkers of chronic prostatitis/chronic pelvic pain syndrome (CP/CPPS). The monitoring biomarkers are progesterone, androstenedione, testosterone, corticosterone, aldosterone, and 11-deoxycortisol. When compared with the mass spectrometry method, all the testing results from the analytes achieved correlation coefficient r values between 0.908 and 0.999 [34]. By applying localised surface plasmon resonance (LSPR) to improve the fluorescent intensity of the dye, the antifouling polymer poly(olygo (ethylene glycol) methacrylate) (POEGMA), Zhou et al. [35] created a microchip which can be inserted into the fabricated portable device to detect PSA in the blood sample. The portable system is comprised of the light source for fluorescence excitation and a smartphone camera to take the fluorescent image for intensity analysis. The LOD was reported to be 100 pg/mL of PSA. In 2017, Mao and co-workers developed C-TBD nanoparticles encapsulating Bis [2,4,5-trichloro-6-(pentyloxycarbonyl)phenyl] oxalate (CPPO) and an AIE photosensitizer of TPE-BT-DC (TBD) to track H_2_O_2_ yielded from tumour cells on mouse models. The peak emission of 660 nm for the yellow colour shows that TBD is an efficient AIE probe to detect tumour cells through imaging. This research claims to have approached the limitation of detection of 2 nM H_2_O_2_ [36]. In 2018, Lippert and Krenek [37] used chemiluminescence probes to detect hydrogen sulphide and hydrogen peroxide in biological specimens, such as saliva, blood, urine, and exhaled breath. A dark box was fabricated which could be attached to the smartphone to take the luminescence image from the mixture of the biomarkers and the fluorophore agents. The design satisfied the general requirements of the portable analysing settings in terms of compact size, rapid processing with smartphone analysis, and high accuracy of results. The biosensors of hydrogen sulphide and hydrogen peroxide are Rhodamine 110 and Rhodamine 6G, respectively. These chemiluminescence probes have LODs of 7.7 µM and 15 µM for hydrogen sulphide and hydrogen peroxide.

### 2.3. Other Biomarker Detection Methods

In 2020, Sun et al. [38] designed a gold nanoarray chip using LSPR to enhance the quantum dot emission for procalcitonin (PCT) antibody detection in sepsis diagnosis. Sun and co-workers fabricated the portable system, controlled by a LabVIEW program, to perform PCT detection on-site at clinics. The LOD of the PCT measurement with the reported system was 0.5 pg/mL [38]. Zhang et al. [39] created a soft microfluidic system which can be attached to a patient’s skin to collect sweat and an analysis was run to monitor kidney disorders. The specific biomarkers were sweat pH, creatinine, and urea concentrations. The results obtained by this colorimetric method were 9 μM to 15 μM for creatinine, 2 mM to 15 mM for the urea, and 4.5–7.5 for pH [39]. Jornet et al. [40] developed a wearable photonic smart band using a surface plasmonic resonance method to collect in vivo signals of the specific biomarkers in the bloodstream. In this system, an implantable biosensor is inserted under the patient’s skin at the wrist, to detect the selective biomarkers in the circulatory system. The outer wrist band is comprised of a nanolaser array system to excite the implantable sensor, which enhances the optical signal from the implant, and an integrated CCD to collect the optical signal reflected from the implant. The wrist band then wirelessly transfers the data to a smartphone for further analysis to monitor the analyte amounts for disease indication [40].

## 3. Portable Device Development

Although recent decades have witnessed revolutionary developments in biomarker detection, undeniably one of the critical factors causing bias, errors, misleading measurements, and wide variations in medical conclusions is the miscommunication among researchers, healthcare staff, and patients [41]. Because of this, device development should take into account the specification that the measurement operation should minimise user error and improve biological data collection and analysis. Hence, some specifications should be considered when reviewing portable device development for biomarker detection, i.e., high sensitivity and selectivity in biomarker detection, the stability of the measurement, rapid measurement, simple operation, and compatibility with different commercial materials or accessories. In most of the measurement designs, there are three parts that cover the whole testing system, i.e., the chemical reaction environment, the luminescence observing area, and the data analysing component. Device development is now presented in terms of three significant components: consumables, hardware, and software.

### 3.1. Consumables

Consumables are accessories for the chemical measurement which support the testing processes, such as cuvettes for containing chemicals, paper strips for absorbing the fluid samples, needles for injecting the solution into containers, and the cap/lid for sealing the chemical inside the containers. The choice of consumables depends on the type of test which can be either a microfluidic paper-based test or a liquid-based test.

#### 3.1.1. Microfluidic Paper-Based Assay

The microfluidic testing platforms mainly follow the principle of the original microfluidic paper-based strip. The microfluidic paper-based strip was first invented by Martin and Synge in 1952, then developed and applied to chemical and biomedical analysis [12,42]. Thanks to the paper strip’s unique porous structure, which is created by pressing cellulosic or nitrocellulose fibres together in multiple layers, fluids can be transported from one point to the other end of the paper strip through capillary structures without any requirement for external forces. This transportation property can help the paper strip absorb and interact with the chosen fluids completely, which is a great advantage in chemical and biomedical analysis. Other enormous advantages are its simple usage, low-cost fabrication, high biocompatibility and biodegradation, and the non-requirement of high standard clinical facilities, proper infrastructure, and well-trained medical staff. This makes paper-based microfluidic platforms one of the best solutions for disease detection and monitoring in developing countries [42]. Nowadays, the technology for manufacturing paper-based microfluidic platforms has been improved to be compatible with modern technologies for portable diagnostic application, and most involve immunoassays, which utilise antibodies to track and detect the biomarkers of interest [43]. Although it has been developed into different forms and applications, the paper-based microfluidic platform still follows the traditional principle of lateral flow [42].

**Figure 2:**
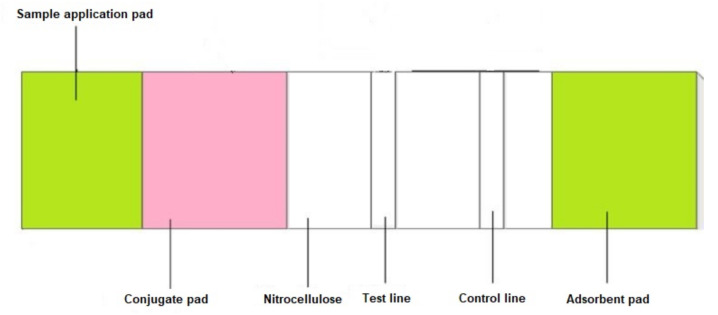
Traditional form of the lateral paper strip [44].

In the lateral flow paper strip, there are four main components: the sample pad, the conjugation pad, the reaction pad, and the adsorbent pad, as shown in Figure 2 [42,44]. (1) The sample is dropped on the surface of the sample pad which is often made of cellulose membrane or glass fibre. The porous structure absorbs and pre-treats the sample due to the specific requirements of the test, such as separating the sample into multiple layers, separating sample components, or removing the interferences [44]. The fluid layers are transported smoothly and continuously through the entire structure of the strip [42,44]. (2) The conjugation pad is comprised of the labelled biorecognition molecules, such as antibodies of latex beads, gold nanoparticles (AuNCs), and so on. Under the capillary effect, the sample fluid is transported from the sample pad to the conjugation pad and the antigen in the sample will react with the conjugate antibody to form the antigen/conjugate antibody complex [42,44]. (3) The antigen/conjugate antibody complex then travels to the reaction pad through the nitrocellulose membrane. In the reaction pad, there are at least two lines of specific antibodies called test and control lines, as shown in Figure 2. The antibodies in the test and control lines can capture the target analytes in the fluid samples and then develop colour to indicate the presence of the biological markers in the samples [42,44]. (4) The absorbent pad plays the role of a sink at the end of the strip. It maintains the flow rate of the solution within the strip, absorbs the residual solution after it flows through the whole structure of the strip, and prevents the solution from flowing back toward the sample pad [44]. All four pads are mounted on a back-support card which is independent of the solution interaction with the cellulose membrane, so it will not affect the flow rate of the fluid, or the reactions between the analytes and the antibodies in the membrane and the sample. Therefore, the back-support card only has the role of securing the positioning of the four pads and assisting with strip handling [44].

There are two methods for observing the paper strip result: the sandwich and competitive strategies methods [43,44]. In the sandwich method, the primary antibody, which captures the biomarkers, is coated at the test line while the secondary antibody, which is against the labelled conjugate antibody, is coated at the control line. When there is no presence of the target analytes (negative), colour only develops at the control line while the test line’s colour does not change. When the target biomarkers are present in the sample (positive), colour develops in both test and control lines [43,44]. In the competitive method, the test line is coated by the antigen, which is the same as the antigen being detected in the sample, and this can bind with the conjugate antibodies. When the analyte of interest is present in the sample, it displaces the antigen in the test line, resulting in the fading of the colour in the test line. Therefore, the principle of paper strip application in diagnosis involves chemical reactions between the biomarkers in body fluid samples and the specific antibodies or antigens equipped in the cellulose or nitrocellulose membranes of the strip, which explains why most of paper strip measurements follow optical detection methods. However, depending on the requirements of the portable testing devices, in the following discussion only the application of paper-based assays with colorimetric, fluorescence, and chemiluminescence are mentioned, as these have been presented in the previous section [45]. As an overview, a summary of the pros and cons for using a paper-based strip in three optical detecting methods is shown in Table 1.

Nowadays, paper strips are manufactured for commercial usage in clinical facilities with a standard design for common testing. This results in a limitation for using commercialised paper strips for detecting biological molecules in the research process. Therefore, researchers have developed microfluidic chips, which still follow the capillary principle of the paper’s porous structure and allow the chemical reaction to stay in its solution state. This new development allows researchers to customize microfluidic paper-based chips to not only satisfy the requirements of their portable devices, but also to ensure the performance of the optical measurement.

**Figure 3:**
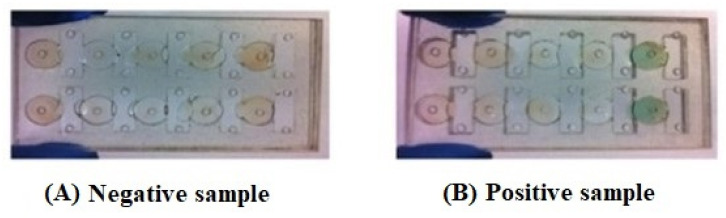
The results of the prostate cancer detection by using the HRP substrate [ABTS (2,2′-Azinobis [3-ethylbenzothiazoline-6-sulfonicacid]-diammonium salt) to track the presence of the prostate specific antigen (PSA) in serum, (**A**) the negative sample (No PSA) where the HRP substrate still stays as a red colour and (**B**) the positive result (PSA presence) where the HRP substrate develops a green colour [46].

For measurement, colorimetric results can be observed by the naked eye, or analysed by software installed on a programming device, such as a computer, laptop, or smartphone. This property is an ideal specification, helping researchers to develop the portable testing system to analyse the colour changes from the chemical mixture [12]. For instance, Adel Ahmed and Azzazy [46] presented a power-free and portable chip that works with solutions using ELISA in monitoring PSA for prostate cancer detection. Magnetic nanoparticles capture the target analytes in serum. Then this complex flows to the region containing the reagents to bind the analytes and the magnetic nanoparticles. This paper-based immunoassay chip performs a sandwich ELISA. When the PSA binds with the horseradish peroxidase (HPR) substrate, a green colour appears on the smartphone camera, as shown in Figure 3, and is then analysed by MATLAB software for RGB intensity per pixel. The limit of detection was reported to be 3.2 ng/mL [46].

**Figure 4:**
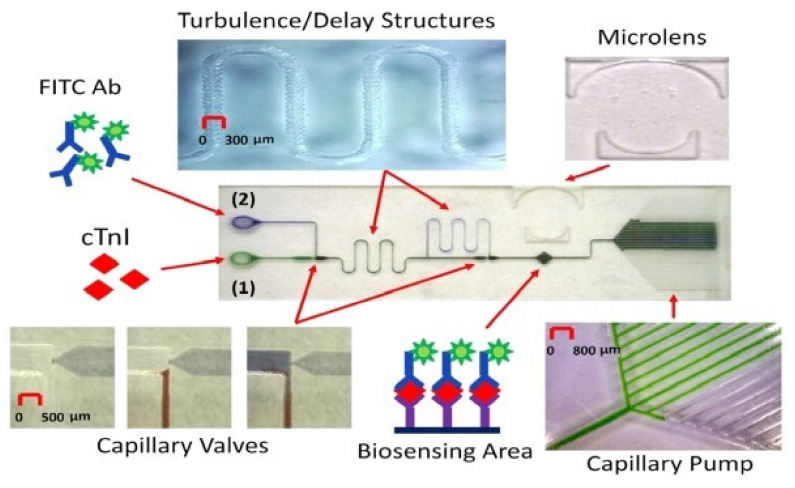
The microfluidic fluoroimmunoassay platform [48].

As mentioned above, for biomarker detection by the fluorescence method, an optical excitation source is needed. This stimulates the analyte/antigen or antibody complex to produce fluorescent light. In 2014, Castro-López et al. [47] reported a portable device with a customed microfluidic chip using the dye fluorescein amidite (FAM) to quantify the tumour necrosis factor (TNF-∝) in human plasma. The fluorescent dye (FAM) is excited by 470 nm blue light and the emission wavelength is 520 nm. The fluorescence light is captured by ESElog, the fluorescence detector (Qiagen), and is then analysed by the program on a computer. The limit of detection for this 3 h measurement was reported to be 3.1 pg/mL [47]. Mohammed and Desmulliez [48] introduced a lab-on-a-chip device which enables the detection of cardiac Troponin I (cTnI), the significant biomarker for monitoring acute myocardial infarction, as shown in Figure 4. The detecting agent is fluorescein isothiocyanate (FITC). A CO_2_ laser is used to engrave the capillary system on PMMA, and a sandwich immunoassay is applied to measure the concentration of cTnI in the blood sample. After mixing the blood sample with a FITC antibody, a 495 nm LED is used to excite the complex. If the cTnI biomarker is present in the blood sample, the complex emits fluorescence with a wavelength of 520 nm which is captured by a photodetector. In this 9 min measurement, the limit of detection was claimed to be 24 pg/mL. Xie et al. [49] designed an AIE-based fluorescent test strip of OPD-TPE-Py-2CN to detect gaseous phosgene, an industrial chemical which can damage the pulmonary alveoli and lead to pulmonary edema, pulmonary emphysema, or death. When combined with phosgene, the AIE probe on the test strip can emit fluorescence and shift the colour from blue to green under the exposure of 365 nm UV light. This research approached the LOD of 1.87 ppm, much lower than the minimum allowed level for human health.

In chemiluminescence detection, the paper-based microfluidic platform has also drawn significant attention from researchers in recent years. Not only do they achieve as high a sensitivity and selectivity as fluorescence methods, but chemiluminescence detection systems also do not require an excitation light source which is indispensable in fluorescence and colorimetric methods. This helps to establish the size and source for portable testing system development [12,26,33]. Wang et al. [50] reported a lab-on-paper device, using sandwich chemiluminescence ELISA (CL-ELISA) and microfluidic paper-based analytical devices (µPADs) to detect α-fetoprotein (AFP), the cancer antigen 125 (CA125), and the carcinoembryonic antigen (CEA) in human serum. This measurement uses luminol-p-iodophenol-H_2_O_2_-HRP-CL as the substrate and showed a linear performance of 0.1–35.0 ng/mL for AFP, 0.5–80.0 U/mL for CA125 and 0.1–70.0 ng/mL for CA. The limits of detection were reported to be 0.06 ng/mL, 0.33 ng/mL, and 0.05 ng/mL for AFP, CA-125, and CEA, respectively. Yang et al. [51] developed an AuNC surface to enhance the chemiluminescence signal in staphylococcal enterotoxin B (SEB) detection to indicate foodborne disease. The luminescent signal is read by a cooled CCD sensor or a plate reader, and the sensitivity of this AuNCs sensor was reported to be 0.01 ng/mL [51].

From what has been mentioned above, the paper-based microfluidic platform has critically advanced the research into biomarker diagnostics. The specifications, which are portability, low-cost fabrication, qualitative or semi-quantitative results, a simple test procedure and a long shelf-life characterize this dry-testing platform development along with the improvement of the portable testing system in recent years. However, there are some drawbacks in the paper-based platform, such as the uncontrolled sample volume consumption, the limit of sensitivity due to the restriction on testing volume, strict requirements in choosing the suitable antibodies and antigens to detect the biomarkers, the dependence of the analysing process on the nature of the sample, the equipment of the antibodies and antigens onto the paper surface, and the methods to secure the stability of the antibodies/antigens after they are attached to the paper surface [12,52]. These drawbacks mean some biomarker detections are preferably processed in solution states of chemical and body fluid samples.

#### 3.1.2. Liquid-Based Detections

**Figure 5:**
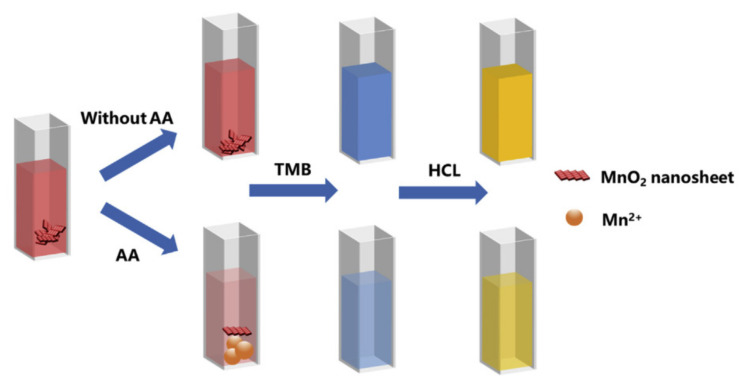
The schematic for ascorbic acid detection using a colorimetric assay with a TMB-MnO_2_ reagent. In the reaction, the serum is injected into the Quartz cuvette, which will be located inside the device for measuring. MnO_2_ is added into the serum sample, followed by the addition of TMB and HCl to shift the solution’s colour from red to blue and yellow respectively [5].

Although operating differently from the paper-based microfluidic platform, liquid-based biomarker detections also follow similar principles in that the obtained results come from reactions between the target biomarkers and the detecting agents. Some liquid-based biomarker detections are now presented for colorimetric, fluorescence, and chemiluminescence methods for portable testing systems. Chen et al. [52] applied ELISA into colorimetric detection to measure bovine serum albumin (BSA) in serum by developing a microfluidic platform, allowing the reagent of 4:1 VHH/horseradish peroxidase (VHH/HRP) to react with BSA under the support of phosphate buffered saline. The developed colour images are captured by a smartphone camera and sent to an Arduino microcontroller to analyse the RGB values in the BSA concentration in the serum sample. The smartphone is used for colour-shifting imaging only, not for signal analysis. Kong et al. [5] reported a smartphone-based microchip for tracking ascorbic acid (AA) in serum. Serum is injected into a quartz cuvette which is located inside the measuring device. MnO_2_ is added to the serum sample, followed by the addition of TMB and HCl to shift the solution’s colour from red to blue and yellow respectively, as shown in Figure 5. The presence of ascorbic acid in the serum enhances the solution’s colour intensity. A smartphone is used to capture colour pictures and customised software is installed on the smartphone to analyse the HSV values, then the correlation between the colour intensity and the AA concentration can be established. The limit of detection for AA in serum is 0.4946 µM.

Coskun et al. [53] developed an albumin tester which benefits from a smartphone’s imaging and computing systems to monitor the albumin level in human urine. Albumin blue 580 fluorescence dye is used as the agent to capture albumin molecules in urine (human serum albumin, HSA). After being uniformly mixed in the test tube, the mixture of fluorescence dye and urine is irritated by a laser diode at a 532 nm wavelength, then a fluorescence of 580 nm is emitted. The intensity of the fluorescence is proportional to the HSA concentration in the urine sample. The LOD of this platform was reported to be 5–10 µg/mL, which is three times lower than the normal clinical range. Akraa et al. [54] used an AIEgen bioprobe, BSPOTPE, to monitor the HSA concentration in urine to indicate the early signs of chronic kidney disease (CKD). A urine sample is mixed with BSPOTPE to produce the testing solution. If HSA is present in the urine sample, the solution can emit a 480 nm fluorescence under the excitation of 340 nm UV, with the intensity proportional to the HSA concentration in the sample. Quimbar et al. [55] introduced a smartphone-based chemiluminescence analyser to detect airway hydrogen peroxide (H_2_O_2_) in exhaled breath condensates. For the solution reaction, 75 µL of an exhaled breath condensate sample is added into 375 µL solution of 3.4 mM BPEA, 3.2 mM imidazole, and 1.9 mM CCPO in 9:1 EtOAc:CH3CN to yield a 500 nm chemiluminescent emission. The limit of detection is 264 nM of H_2_O_2_ in human exhaled breath condensates, and the analysing process is performed by an app installed on a smartphone.

### 3.2. Hardware Support and Analysing Software

In portable testing systems, while consumables are the components supporting the chemical reactions, solid support accessories are needed to assist the measurement. These provide a controlled-condition environment for the measurement, secure the positioning of different components, provide the excitation elements for specific tests, capture the resulting signals, analyse the data, or transfer the data to external instruments. Many researchers have used smartphones as powerful assistants in the design of portable medical analysis devices. With their high-resolution CMOS cameras and a range of settings such as white balance, auto-focus, custom ISO value, as well as their efficient computing processors, high battery capacity and wired or wireless connections, such as Bluetooth, Wi-Fi, internet, etc., smartphone-based medical devices have been upgraded to monitor human health conditions and to detect the micro signs of chemical or biological compounds [56]. Some smartphone-based medical detectors are integrated with customised software to run the analysis themselves while others only capture the testing signal and transfer it to an external instrument for further analysis. In this report, we focus on portable devices using optical detection to monitor biomarkers in body fluids. The major areas of interest are the developments in portable testing systems using colorimetric, fluorescence, and chemiluminescence detection.

#### 3.2.1. Colorimetric

As explained above, colorimetric detection is based on colour changes or colour intensity generated by the chemical reactions between the biomolecules of interest and the detecting agents. The primary portable device design should consist of three major parts: the chemical reaction carrier, the image capture system, and the analysing components. Shen et al. [57] introduced a smartphone-based device to quantify the chromaticity values from pictures of pH levels in commercial colorimetric paper test strips. This hand-held colorimetric analyser uses the CMOS camera of HTC and Blackberry smartphones to capture these images, then sends those images to computing devices to run MATLAB for colour intensity analysis. One of the problems in using a smartphone as a colour photo detector stems from the camera’s auto-functional settings. To compensate for this, the reference colour chart of 12 known colour intensities with seven greyscale regions and five colour regions ranging from blue to red is used. This reference chart shows potential to minimise the effects of the smartphone’s camera auto-settings and ambient light changes. The LOD of this design was claimed to be 0.5 in pH value. Wang et al. [24] developed a smartphone-based system running with a customised microchip ELISA to track the presence of the HE4 biomarker in urine to indicate ovarian cancer. When the molecule detector TMB binds with biomarker HE4 in urine, a blue colour develops in the solution, and the 3.2-megapixel camera on the smartphone (Sony Ericson i790) captures this shifting colour in pictures. An app installed on the smartphone analyses these pictures to obtain the RGB pixel values. The red pixel value is chosen to estimate the HE4 concentration in the urine sample. The LOD of this cell phone-based device was claimed to be 19.5 ng/mL.

Oncescu et al. [58] presented a smartphone-based prototype using colorimetric detection to monitor the pH values of sweat and saliva. The pH of sweat is proportional to the sodium concentration, which is used in muscle cramp detection. In contrast, the pH of salvia is examined to track enamel decalcification, the specific biomarker for monitoring the breakdown of calcium in the teeth. The customised strip is comprised of three components: a 9 mm × 4 mm piece of a Hydrion Spectral pH indicator for sweat testing (ranging from 5.0 to 9.0) or saliva testing (ranging from 1.0 to 14.0), a 9 mm × 4 mm piece of a known colour value, working as the reference for calibration, and a polydimethylsiloxane (PDMS) diffuser for uniformly distributing the camera flash to the testing paper sections. All components are covered in a black case fabricated by 3D printing with Vera black material to eliminate the effects from external light and to keep a suitable distance between the camera and the test strip. An app for an iPhone was also developed to run the colorimetric analysis by examining the hue value from hue-saturation-luminance analysis (HSL) to give the quantitative pH results.

In summary, as shown in Table 2, most developed devices using the colorimetric method in biomarker detection have a similar mechanism: the primary light source uses white light, which can be achieved by using the flashlight from the camera through an equipped diffuser. For the chemical reaction support, prominent researchers prefer using a microfluidic paper-based assay to reduce the device’s size and to broaden the observed section where the colour changes, which supports the image capturing process. The image processing mainly works on the analysis of RGB or HSL values. A critical aspect of portable device design is the solid support component which carries the whole system and secures the position of every component, as well as providing a suitable environment for running measurements. Finally, alongside the development of digital and computing systems, the portable system is constantly being upgraded to integrate the analysing software into portable devices, which reduces the testing time as well as improving the quality of the biomarker measurement.

#### 3.2.2. Fluorescence

Unlike colorimetric methods, devices using fluorescence detection require a specific excitation energy source to activate the fluorophores to turn them into luminescent components. Hence, researchers must use a suitable light source to obtain the most benefit from the fluorophores’ properties, depending on the requirement of excitation for various compounds. However, similar to colorimetric detection, smartphone technology is beneficial in most of the portable fluorescent bio-signal measurements. Either the smartphone is used as signal collector only and transfers the received signal to an external equipment for analysis, or integrated software will process the data on the smartphone. Mudanyali et al. [62] designed a cell phone-based rapid diagnostic test (RDT) reader platform, using fluorescent imaging and an analysis of commercial lateral flow tests to monitor malaria, HIV, and tuberculosis (TB). Two Samsung Galaxy S II smartphone-based prototypes were designed: one uses two AAA batteries to supply power for the LED source while the other draws energy from the smartphone through the USB port. In the test operation, lateral flow tests are applied to a blood sample before insertion into the prototype. The LEDs are arranged in three rows, providing the excitation wavelength of 565 nm in two ways: two LED arrays are attached under the RDT tray for reflection imaging; and one LED array is located at the top of the RDT tray for transmission imaging. Depending on the test requirement, users can trigger the physical switch on the prototype to choose the suitable imaging method. The phone’s camera takes the raw fluorescent images of the test and control lines on the RDTs. These images are processed by an app on the smartphone with a customised algorithm to generate the quantified result of the antigen density from the test line’s colour intensities [62]. In this design, researchers created an isolated area for fluorescent imaging to minimise the effect of the external environment, which secures the stability and repeatability of the measurement. Moreover, the patient’s information and the testing results can be stored in the internal memory of the smartphone or uploaded to the internet through wireless communications for further analysis. 

Coskun et al. [53] developed a Samsung smartphone-based analyser to detect albumin in human urine for CKD indication. This albumin tester is comprised of a 148 g attached support, a two AA battery powered laser diode for light excitation of a 532 nm wavelength, two cuvettes of 6 mm × 2 mm × 15 mm for storing the urine sample and the fluorescent dye, an android smartphone built-in camera for fluorescent imaging, and an app installed on the same phone for image processing to give the quantitative result. All measurement components are secured in a solid support, which is designed in Inventor software and fabricated by 3D printing. The LOD was claimed to be 5–10 µg/mL, three times lower than the normal range for clinical acceptance (the albumin level should be lower than the threshold of 30 µg/mL in human urine) [53,63]. Two customised cuvettes are used, one for controlling and the other for testing. The control cuvette is wholly filled with a fluorescent dye (albumin blue 580) while the test cuvette is partially filled with the dye, giving space for the urine sample to be added later. A plano-convex lens and a fluorescent interference filter are arranged in the middle of two tubes with the smartphone camera to collect the fluorescent emission and to filter out the scattering excitation light, respectively. After being captured by the CMOS camera of the smartphone, fluorescent images of both tubes are processed to produce the optical values of the test tube ($I_{Test})$ and the control tube ($I_{Control}$). Then, the app uses these values to generate the relative fluorescence unit ($RFU=\frac{I_{Test}}{I_{Control}}$) and calculate the concentration of albumin in the testing sample.

In 2020, Huang and co-workers developed a coffee ring test kit using an AIE bioprobe of TPE-TS@Eu/GMP ICPs to detect dipicolinic acid (DPA), a biomarker for Bacillus anthracis (B. anthracis) spores, which cause anthrax after gemination. A smartphone is used to take fluorescent images of the coffee ring under the excitation of a 405 nm UV lamp. The image processing focuses on analysing the colour pixel in the blue channel (430–500 nm) and the red channel (580–650 nm). On the smartphone, the software Image J analyses the red and blue values, then generates the ratio between the red and blue (I_Red_/I_Blue_). When the DPA level increases, the blue area on the coffee ring will extract, leading to the weakening of the blue signal. Meanwhile, the red area on the coffee ring expands making the red colour brighter. Following this methodology, evaluating the red/blue ratio will give a quantitative result of the DPA level in the biological sample. The detection limit of this coffee ring sensor was claimed to be 27 nM [64].

Most developed devices using the fluorescence method in biomarker detection have a similar mechanism, as shown in Table 3, in that the smartphone is used as the image capture component. Few researchers use a CCD camera while some use a photodiode to measure the optical values from the luminescence. However, the CMOS camera has gained more attention in portable system design. Consequently, although some researchers use a CCD camera to obtain a better resolution in the capture of optical images or apply a photodiode to evaluate the visual numeric data, the majority use smartphones integrated with a CMOS camera to support the portable testing systems, helping to simplify the hardware design, as well as minimizing the device’s cost.

The luminescence source is also a point to be considered as it affects the whole system’s operation. There are three types of luminescence sources for such devices: the lamp, the LED, and the laser diode. The LED and laser diode have some similar characteristics (as shown in Table 4) and are more prevalent for portable device development than the powered lamp. Depending on whether a scattered light or a strongly focused light beam is needed, LEDs or laser diodes, respectively, are employed.

Fluorescence detection requires a specific light source so the power supply should not be overlooked. Opinions vary about using an external power supply (adapter or battery assembly) or drawing energy from integrating the smartphone through an audio output jack or charging input plug. However, harvesting power from a smartphone to supply the luminescence source has some obstacles: (1) it requires a complicated converter and an amplifier because the obtained current from the audio output jack or the charging input plug on the smartphone is significantly low and (2) the smartphone’s USB charging port is specially manufactured with an integrated chip to protect the smartphone from overcharging damage, so the power going through this port is limited. Hence, drawing power from a smartphone’s battery to run the luminescent source seems to be impractical compared with using an external battery or a charging adapter. In fact, most developed portable devices use a battery or an outlet adapter to supply power for electronic components. Each solution has advantages and disadvantages: the battery can efficiently satisfy the portable requirement, but its power will reduce over time, which requires an indicator or a regulator component to manage the power supply from the battery. In contrast, the outer adapter has its own regulating component, which can guarantee the stability of the power supply to the device. However, the cons for using the adapter are that it requires the power source and will affect the portability specification of the device. Depending on the requirements of the portable device, the suitable power supply will be considered. Most developed devices employ filters in the optical measurement process. In optical measurements, there are uncountable confounding factors, such as the effect of external light, the light degradation of the luminescent source, the reflecting or scattering of light inside the optical evaluating room, or the noise from the chemical components themselves, which may add a different light wavelength into the data collection. Using filters helps distinguish these noises from the preferred signal. This also reduces the complexity in designing the analysing component, resulting in a reduction in the size and cost of the device. 

Simultaneously, to ensure the precision of the testing system, quality control or calibration are indispensable components for all developed portable devices. There are two opinions about the better method for operating the calibration: running periodic calibration and running calibration in every sample test; in other words, embedding the calibration step into the device, so that whenever users perform a single test, two results occur: one for the new test and one for the calibrated value. Both methods have advantages and disadvantages, and researchers select one according to the requirement for the precision of the test or the design’s simplicity. In this, consideration is given to the measurement results’ liability, and the device’s portability and cost. In summary, portable devices using a fluorescence method to detect biomarkers in body fluids are shown in Table 5. 

#### 3.2.3. Chemiluminescence

**Figure 6:**
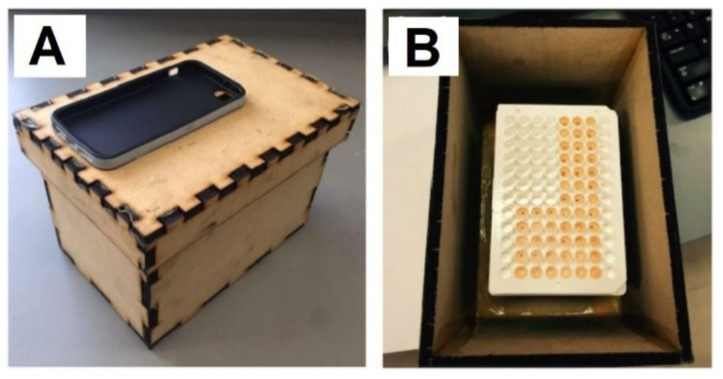
(**A**) The overview of the wooden dark-box with the iPhone case attached on the top so that the phone’s camera can take a picture of the solution plate inside. (**B**) The 96-well plate containing the detecting reagent and the sample is placed inside the box for taking chemiluminescent images [55].

As indicated earlier, in chemiluminescence detection, testing devices require no light source for the excitation of the luminescent properties. This helps simplify the design and structure of such portable devices. Quimbar et al. [55] developed a smartphone-based dark box, using a diagnostic platform to monitor airway hydrogen peroxide (H_2_O_2_) using chemiluminescence. An exhaled breath condensate sample of 75 µL of is added into 375 µL solution of 3.4 mM BPEA, 3.2 mM imidazole, and 1.9 mM CCPO in 9: 1 EtOAc: CH_3_CN to yield a 500 nm chemiluminescent emission. The luminescent picture is captured by the phone’s built-in CMOS camera and a customised app on the phone runs intensity pixel analysis, then indicates the concentration of H_2_O_2_ in the sample. The dark box is 5 × 7.5 × 5.5 inches and is attached to the smartphone case to secure the positioning of the camera and the 96-well plate located inside the box, as shown in Figure 6. The LOD of this platform is 264 nM of H_2_O_2_ in human exhaled breath condensates [55].

Not all developed smartphone-based biomarker detecting devices can analyse the obtained signal themselves; some need an external device. Kim et al. [70] designed a 3D-printing holder to secure the positioning between a smartphone and a solution tube for chemiluminescent imaging in the quantitative measurement of the reporter bacteria OD600. The 490 nm luminescent images were captured by the smartphone’s camera then they were wirelessly transferred to the external PC for analysis by MATLAB. Kim and co-authors compared the performance of different brands, such as the iPhone 5S, Samsung Galaxy S4, Samsung Galaxy Note 3, Oneplus One, and LG G2, and found that the measurement using Oneplus One showed the best the limit of detection of 7.9 × 10^6^ CFU/mL. Table 6 shows some research using the chemiluminescence detection method with the developed portable biomarker detection devices.

## 4. AIE Related Research

Since it was first reported in 2001 [74], AIE has drawn much attention from researchers in biomarker detection [75]. AIEgen probes have enhanced the performance of measurements in terms of sensitivity, accuracy, selectivity, and stability with instinct aggregation induced emission features. Although uncountable reports have been presented of using AIEgens in biomarker monitoring, reports for portable AIEgen-based devices are limited. There are opportunities here for multidisciplinary researchers to develop the AIEgen bioprobes embedded with portable devices. In this section, a short summary of the highlighted research on developing a portable system using AIEgens to detect biological analytes is discussed.

**Figure 7:**
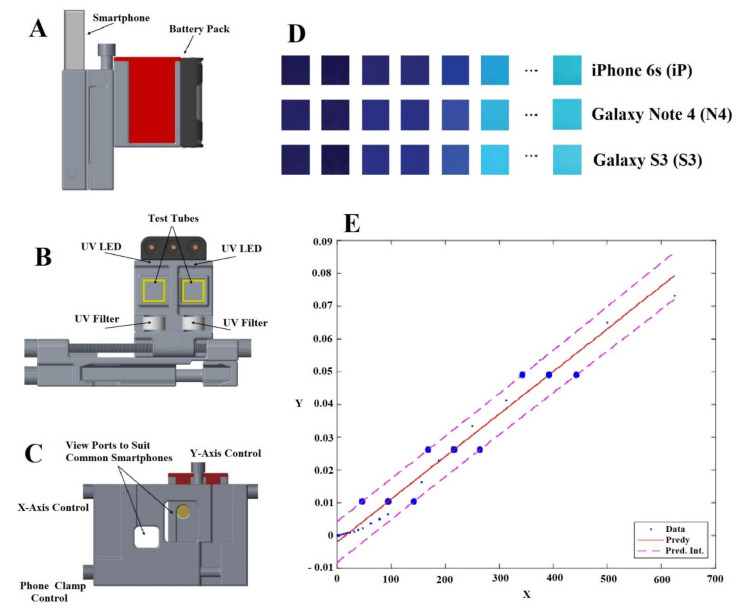
(**A**) external and (**B**) internal design of imaging housing attached to a smartphone and (**C**) adjustable viewports and clamp controls for the AIEgen-based 3D printing device. (**D**) Images of different HSA concentration levels taken by 3 smartphones after the AIEgen, BSPOTPE, was added. (**E**) Linear prediction model with internals with the *x*-axis of HSA concentration and the *y*-axis of light intensity of the AIEgen probe after it was combined with HSA [54].

Following the general structure of a biomarker detecting device, the developed system consists of the consumable components which are various AIEgens, the solid supporting part, and the signal analysing software. Several AIEgen-based portable devices have been reported in recent years. In 2018, Akraa and co-workers reported the uTester, the smartphone-based prototype using a commercially available AIEgen, i.e., BSPOTPE to detect HSA in urine samples. A preliminary evaluation of the device combined with the AIEgen has confirmed the effectiveness of the proposed solution and the viability of such a smartphone-based device for people who have already developed or are prone to CKD to regularly perform portable urine testing in order to self-monitor their own health conditions without the burden of frequent visits to their doctors. Those results are as shown in Figure 7 [54]. In 2020, Ramasubramanian’s group presented a novel AIE probe, the anthracene-conjugated imidazo[1,5-a]pyridine (TL19), and a three dimension (3D) printed prototype which can track copper (Cu^2+^) presence in an aqueous medium. This prototype supports an android smartphone to take fluorescent images of the reaction between Cu^2+^ and TL19. Then, the software DAS-6 installed on the smartphone will analyse the RGB values to give the result of Cu^2+^ presence in the sample with an LOD of 9 pM [69]. Chen et al. [76] firstly developed a fluorescent paper analytical device (FPAD), using nanoparticles (PTDNPs-0.10) and two-dimension MnO_2_ nanoflakes (2DMnNFs) to monitor the existence of organophosphorus pesticides (OPs). This device uses a smartphone to capture the variation in fluorescent intensity, and a colour recogniser will analyse the RGB values to give an indication of the OPs level. This device can approach the LOD of 0.73 ng/mL [76]. In another report in 2020, Huang’s research group introduced the coffee ring test kit, which uses TPE-TS@Eu/GMP ICPs to capture DPA analytes and to give an indication of the presence of B. anthracis spores. A smartphone was used to take fluorescent images from the paper ring test kit when it was in contact with a sample containing DPA under the exposure of a 405 nm UV lamp. The Image J software installed on the smartphone analyses the red and blue values of the reaction section to give an indication of the DPA levels in the sample. These reports show that portable devices with POC analysis features are interacting with the emerging AIEgens bioprobes by multidisciplinary researchers to form AIEgen-based portable devices to detect and monitor biomarkers in body fluids.

Meanwhile, researchers have claimed that the reported AIEgens have great potential to associate with portable devices to form POC testing systems for the detection and monitoring of biomarkers in body fluids. For example, an AIE-based fluorescent test strip of OPD-TPE-Py-2CN was designed by Xie and co-workers to detect gaseous phosgene. Although they did not show the design of the portable supporting device, the researchers introduced a non-laboratory method for detecting gaseous phosgene [49]. In the same year, Zhang’s research group reported an assay using TPE-HPro to detect L-Lactate oxidase (LOx) in aqueous fluid. This research can be used as a solution to apply AIEgen into L-Lactate detection in saliva and urine samples [32]. In 2019, Yu and co-workers succeeded in preparing a portable test strip embedded with the zinc-based metal-organic framework of pyromellitic acid (Zn-BTEC), which can detect chlortetracycline (CTC) at the low limit of 28 nM [77].

## 5. Discussion and Future Trends

The rapid development of mobile technology has promoted its application in human health monitoring in modern society. Although optical methods for biomarker detection have varied greatly over the last decades, applying the powerful processing from smartphones has brought numerous advantages in detecting disease markers in body fluids. Such detection gives early indications for critical diseases and informs the modification to suit different stages of treatment. Smartphone usage for this purpose satisfies the requirements of portable healthcare device design, such as low cost, simple usage, rapid result analysis, accessible communication between patients and healthcare staff, and high reliability.

However, drawbacks still exist in designing the consumables and choosing the computing processor which require further consideration. Firstly, the choice of a paper-based testing platform or a fluidic-based testing platform depends on the compatible biomarker detecting probes and the measurement conditions. The paper-based microfluid platform has undoubted advantages in biomarker diagnostics in terms of high portability, qualitative or semi-quantitative results, a simple test procedure and a long shelf life in suitable storage conditions. Nevertheless, its limitations are also significant. The uncontrolled sample volume absorption and nonuniform chemical distribution on the paper surface results in a low sensitivity. The strict requirements for suitable capturing agents for biomarkers leads to the limitation in the detection of some disease types. The integration of biological molecule detecting agents onto the paper surface raises some obstacles for paper-based testing platform fabrication. In contrast, the fluidic-based measurements may overcome the drawbacks of biomolecule detection in paper-based testing methods. However, liquid-based testing encounters trouble in dealing with solution’s stability depending on the environment and time of storing, is easily cross-contaminated, and requires a training process for chemical preparation.

Secondly, although smartphones benefit the portable system design with a high-resolution camera, excellent mobility, powerful computing processor and they are being improved along with the development of modern society, numerous drawbacks exist. The smartphone’s built-in cameras used for capturing the optical signal yielded from chemical reactions have their setting functions, such as auto-focus, auto-white balance, auto-ISO and auto-filter, which supports the users’ daily activities, but affects the optical result in chemical testing for biomarker detection. Some researchers prevented these manufacturer’s functions from affecting results by turning off the auto-functions of the camera, or by choosing the specific settings for running the test, or by using a commercial or customised app to control the camera settings. This issue has meant that not all brands of smartphones are applied for biomarker detection systems. With android phones, users are allowed to interfere with the camera’s customised system. Moreover, due to the wide range of optical bio-signals, some smartphones cannot be integrated with the analysing software to process the obtained data. The evidence is that in many studies, smartphones only collect the optical signal, then transfer it to an external computing system for analysis. This results in the whole design becoming bulky and complicated to manufacture.

Finally, the rapid roll of technological development forces smartphones to be upgraded in terms of integrating more imaging functions, changing the imaging processor, expanding the phone’s dimensions, raising their price, as well as replacing previous smartphone generations. These changes also contribute to the burden on the research and application of smartphones for mobile medical surveillance systems.

To deal with the obstacles mentioned above, some solutions have been applied in the development of portable biomarker detectors. First, the cost-effective manufacturing technology of 3D printing shows potential to reduce fabrication costs in prototyping the devices. The widespread use of 3D printing in the market has helped this fabricating method become more feasible for users and researchers. Besides, its high flexibility allows researchers to take the most benefit from the combination of other technologies, such as the smartphone, CCD camera, ultraviolet light source, or customised microfluidic paper-based platform test strip. Secondly, for the data analysis, currently researchers have applied machine learning to data collection steps, and have also used the digital cloud to store the obtained data. This not only helps with the data processing of projects in operation, but it also establishes a database as a premise for long-term research in the future. Thirdly, soft polymers, gels and hydrogels dominate modern biomaterials and have gained attention from researchers for decades. These technologies can also be used as chemical carriers which can store the detecting reagents to increase their shelf life, or create a controlled environment for chemical reactions, as well as ensuring the stability of these chemicals. Although challenges still exist in designing portable medical devices and further research is needed, portable biomarker detectors have a promising future that will enable significant improvements in our health monitoring standards.

## 6. Conclusions

In conclusion, the development of bioprobes and portable devices for detecting and monitoring biomarkers in body fluids has been rapidly growing in recent years. Due to the limitation of this review, only some primary optical measurement methods applied in portable systems for detecting biomarkers from body fluids have been presented. Although drawbacks are still evident, the contribution of mobile technologies in the POC analysing system design has a limitless potential for further development. Researchers can take advantage of the evolution of modern technology and combining with emerging bioprobes, such as AIEgens and others to improve the standards of POC medical devices. Furthermore, designing portable biomarker detection devices, which minimizes the burden of needing high standard medical testing instruments for the same purpose in hospitals, helps enable access to swift diagnosis for patients in rural areas and those with limited time and resources to visit clinical facilities for routine health checks.

## Figures and Tables

**Table 1:** Summary of the microfluidic paper-based strip applied in three optical detecting methods: [44].

	Biomarker Agents	Pros	Cons
Colorimetric	Colloidal gold nanoparticles and others.	Optical properties are dependent of the particles’ size and shapes, which are controllable. Friendly to environment. High affinity to biomarkers. High stability with long lasting time.	High selectivity to specific biological markers.
Fluorescence	Fluorophores. e.g., rhodamine, AIEgens	Higher sensitivity than colorimetric. Fluorescent intensity can be controlled by managing the excitation light source.	Chemical and metabolic degradation results in short time storage.
Chemiluminescence	Enzymes, AIEgens and others	High sensitivity compared to colorimetric and fluorescence.	Rapid reaction and degradable intensity in the final mixture require highly sensitive sensors. Short time storage.

**Table 2:** Portable devices using the colorimetric method to detect biomarkers in body fluids.

Ref	Biological Information	Dry or Liquid Based Testing	Smartphone or Other Devices	Light Source	Data Collecting	Data Analysing	Result
[57]	Detect pH level in Urine	Paper test strip	Smartphone (HTC and Blackberry)	White light (5000 K)	Smartphone (CMOS camera)	Computer (MATAB)	LOD: 0.5 in pH value.
[59]	Monitor Dengue and Chikungunya Viral infections.	Paper test strip.	Customed device.	White light (7500 K)	Raspberry Pi CMOS image sensor module	Computer (MATAB)	Consistent and accurate in detecting dengue and chikungunya in 30 min.
[60]	Detect alpha-fetoprotein and mucin-16 in serum	Customed paper strip.	Smartphone (iPhone 7).	White light (Sunlight).	Smartphone (CMOS camera).	Computer (ImageJ software).	LOD: 1.054 ng/mL for AFP; and 0.413 ng/mL for MUC16.
[52]	Detect bovine serum albumin in serum.	Customed microfluidic chip.	Smartphone (NA brand).	NA.	Smartphone (CMOS camera).	Arduino microcontroller (Customed software).	The system can immediately present the analysis result of BSA concentration in serum.
[24]	Detect Human epididymis protein 4 biomarker in Urine	Customed microfluidic chip ELISA	Smartphone (Sony Ericson i790)	White light	Smartphone (3.2-megapixel CMOS camera)	Computer (MATAB).	LOD: 19.5 ng/mL of HE4.
[58]	Detect Sodium concentration and Enamel decalcification in Saliva	Customed paper strip.	Smartphone (iPhone4 and iPhone 4S)	White light (Camera flash)	Smartphone (CMOS camera))	Smartphone (Customed app)	Detect the risk of dehydration and evaluate the impact of dietary changes on the risks of enamel decalcification.
[5]	Detect ascorbic acid in serum.	Liquid test.	Smartphone (Vivo X7).	Customed light source.	Smartphone (CMOS camera).	Smartphone (Customed app).	LOD: 0.4946 µM for AA in serum.
[61]	Measure nucleic acid	Customed paper strip.	Smartphone.	Smartphone’s flashlight	Smartphone (CMOS camera).	Smartphone (Commercial app).	LOD: 0.39 fg/µL for RNA in serum.

**Table 3:** Comparison of the pros and cons of CCD and CMOS cameras and a photodiode.

Camera	Data Type	Resolution	Power Required	Popularity for Researching	Cost	Electronic Support
CCD	Digital	High	High	Medium	High	High. Require electronic circuits to control the camera, to regulate power supply, to convert analog-digital signal, etc.
CMOS	Analog	Medium	Medium	High	Medium	Low. Only power supply circuit required.
Photodiode	Numeric	Low	Low	Low	Low	Medium. Require power supply circuit and the signal detecting circuit.

**Table 4:** Comparison characteristics of an LED, a laser diode and a lamp for the luminescence source of a portable device.

Light Source	Size	Power Required	Intensity	Light Distribution	Popularity for Researching	Cost	Electronic Support
LED	Small	Low	Medium	Scattering	High	Low	Low, only power supply circuit required.
Laser diode	Small	Low	High	Focus beam	High	Low	Low, only power supply circuit required.
Lamp	Large	High	Extremely high	Scattering	Low	High	High, require complex power supply circuit.

**Table 5:** Portable devices using the fluorescence method to detect biomarkers in body fluids.

Ref	Biological Information	Dry or Liquid Based Testing	Smartphone or Other Devices	Excitation Wavelength	Fluorescent Emission	Data Collecting	Data Analysing	Result
[65]	NA.	Customed solution test tray.	Smartphone (Nokia 1020).	465 nm.	Depending on the conjugated reagent.	Smartphone (CMOS camera).	Computer (Commercial app).	LOD: 80 fluorophores/diffraction-limitted spot.
[32]	Chloride in sweat	Solution test.	Smartphone (HTC One M9).	365 nm.	441 nm.	Smartphone camera.	NA.	LOD: 0.8 nM of chloride in sweat.
[62]	Malaria, HIV and tuberculosis (TB) in whole blood.	Commercial paper test strips for each disease.	Smartphone (Samsung Galaxy SII)	565 nm.	Depending on the disease detections (NA)	Smartphone (CMOS camera)	Smartphone (Customed app)	Correctly and qualitatively detects the infected patients.
[53]	Albumin in urine	Solution test.	Smartphone (Samsung Galaxy SII)	532 nm.	NA	Smartphone (CMOS camera).	Smartphone (Customed app)	LOD: 5–10 µg/mL
[66]	Concentration of chloride, sodium and zinc in sweat.	Customed test strip.	Smartphone (iPhone 6 Plus).	451 nm.	511 nm for chloride. 515 nm for sodium. 519 nm for zinc.	Smartphone camera.	Smartphone (Customed app)	LOD: 28 mM for chloride; 36 mM for sodium; and 3.6 µM for zinc.
[54]	Albumin concentration in urine	Solution test.	Smartphone (iPhone 6s, Samsung Galaxy Note 4, and Galaxy S3).	340 nm.	~ 480 nm.	Smartphone (CMOS camera).	Smartphone (Customed app)	Quantitatively detects albumin in urine.
[67]	E. coli K12 in urine.	Customed microfluidic chip.	Smartphone (iPhone 6S).	NA.	NA.	Smartphone (CMOS camera).	Smartphone (Customed app).	LOD: 240 CFU/mL for E. coli K12 in urine.
[68]	Human chorionic gonadotropin in urine.	Customed paper test strip.	Smartphone (iPhone 5S).	NA.	NA.	Smartphone (CMOS camera).	Smartphone (Customed app).	LOD: 45 pg/mL for hCG
[64]	Dipicolinic acid from Bacillus anthracis spores	Customed AIEgen-based paper coffee ring.	Smartphone	405 nm.	Blue (430–500 nm). Red (580–650 nm)	Smartphone (CMOS camera).	Smartphone (Image J).	LOD: 27 nM for DPA.
[69]	Detect Cu^2+^ in aqueous sample	Solution test	Raspberry Pi-ARM V11	365 nm	510 nm.	Raspberry Pi camera 5 MP	Software DAS-6 from IBH to analyse RBG.	LOD: 9 pM of Cu^2+^ ion.

**Table 6:** Portable devices using the chemiluminescence method to detect biomarkers in body fluids.

Ref	Body Fluid	Dry or Solution-Based	Smartphone or Other Devices	Luminant Emission	Data Collecting	Data Analysing	Result
[70]	Reporter bacteria OD600	Solution	Smartphone (OPO OnePlus One)	490 nm	Smartphone (CMOS camera).	Computer (MATLAB)	LOD: 7.9 × 10^6^ CFU/mL
[55]	Airway hydrogen peroxide in exhaled breath condensates.	Solution.	Smartphone (iPhone 6).	500 nm	Smartphone (CMOS camera).	Smartphone (Customed app)	LOD: 264 nM of H_2_O_2_ in human exhaled breath condensates.
[71]	Total bile acids and total cholesterol in serum.	Solution.	Smartphone (iPhone 5S).	NA.	Smartphone (CMOS camera).	Smartphone (Commercial app).	LOD: 0.5 µmol/L for total bile acids and 20 mg/dL for total cholesterol in serum.
[72]	Salivary cortisol.	Paper test strip.	Smartphone (NA smartphone’s brand).	NA.	Smartphone (CMOS camera).	Smartphone (Customed app).	LOD: 0.3 ng/mL.
[73]	Prostate specific antigen in serum.	Customed microfluidic solution chip.	Smartphone (NA smartphone’s brand).	NA.	Smartphone camera.	Smartphone (Customed app).	LOD: 0.1 ng/mL.
